# Ab-initio Study of structural, elastic, electronic and optical properties of hexahalometallate single crystals K_2_XBr_6_(X = Se, Pt)

**DOI:** 10.1038/s41598-022-12570-1

**Published:** 2022-05-18

**Authors:** Y. Naceur, H. Bourbaba, M. A. Ghebouli, L. Krache, B. Ghebouli, T. Chihi, M. Fatmi, Sultan Alomairy

**Affiliations:** 1 LPDS University of Tahri Mohamed, 08000 Béchar, Algeria; 2 Department of Chemistry, Faculty of Technology, University of Mohamed Boudiaf, 28000 M’sila, Algeria; 3 Research Unit On Emerging Materials (RUEM), University Ferhat Abbas of Setif 1, 19000 Setif, Algeria; 4 PQSD Laboratory, Department of Physics, Faculty of Science, University Ferhat Abbas of Setif 1, 19000 Setif, Algeria; 5 Laboratory of Studies Surfaces and Interfaces of Solids Materials, Department of Physics, Faculty of Science, University Ferhat Abbas of Setif 1, 19000 Setif, Algeria; 6 Department of Physics, College of Science, Taif University, P.O. Box 11099, Taif, 21944 Saudi Arabia

**Keywords:** Materials science, Physics

## Abstract

Some physical properties of hexahalometallate K_2_XBr_6_(X = Se, Pt) were computed in the zinc blend structure using GGA-PBESOL. The cell constant of K_2_SeBr_6_ and K_2_PtBr_6_ is consistent to the experiment value quoted in the literature, where the error is 0.95% and 1%. K_2_SeBr_6_ and K_2_PtBr_6_ present covalent bonding, high anisotropy and are ductile. The elastic constants of K_2_SeBr_6_ and K_2_PtBr_6_ are significantly smaller due to their larger reticular distances, lower Coulomb forces and then they are soft and damage tolerant. The interatomic separation is greater in K_2_SeBr_6_ than in K_2_PtBr_6_, hence the Coulomb interaction in K_2_PtBr_6_ is greater than that of K_2_SeBr_6_. The internal coordinate of Br atom in K_2_PtBr_6_ is lower than that of the same atom in K_2_SeBr_6_, and this can be explained by the fact that it is inversely proportional to the atom radius of Se and Pt. There are two major plasmonic processes, with intensities 3.7 and 1.35 located around 53.5 nm and 72.8 nm for K_2_SeBr_6_ and K_2_PtBr_6_.

## Introduction

Progress in experiment and theory, coupled with computational model is accelerating the discovery of new materials with useful physical parameters. The cubic antifluorite class K_2_XBr_6_(X = Pt, Se) have received increased interest since they exhibit structural phase transitions at lower temperatures. The family of hexahalometallate attracts researchers due to their light-absorbing materials in photovoltaic applications. The hexahalometallate double perovskites K_2_XBr_6_(X = Pt, Se) crystallize in the cubic antifluorite K_2_PtCl_6_ structure. They have the stoichiometric formula X_2_MA_6_, where X, M and A are alkaline metal, polyvalent or heavy transition metal and halogen. The K atom in K_2_XBr_6_ (X = Pt, Se) of the three-dimensional structure is bonded to twelve equivalents Br atoms to form KBr12 cuboctahedra. The faces contain six equivalents KBr12 cuboctahedra and four equivalents PtBr6 (SeBr6) octahedral. Studies conducted by other researchers, it is stated that, the investigation on elastic constants and compressibility of K_2_XBr_6_(X = Pt, Se) has carried out experimentally by N. Wruk et al. using Brillouin scattering and ultrasonic wave velocity measurements^1^. The study conducted by Walter Abriel and Mary Anne White on K_2_SeBr_6_ by x‐ray powder diffraction in the temperature range 10 K to 290 K, and heat capacity measurements indicates three phases for K_2_SeBr_6_, K_2_PtCl_6_ cubic structure, Rb_2_TeI_6_ tetragonal structure and K_2_TeBr_6_ monoclinic structure^2^. The phase-transition temperatures of hexahalometallate material K_2_PtBr_6_ (K_2_SeBr_6_) have been studied experimentally and found to be 209 K, 221 K and 249 K^1^ (78 K, 105 K, 137 K, 143 K and 169 K^1^). K_2_XBr_6_(X = Pt, Se) hexahalometallate materials show a suitable energy gap, sufficient absorption, low reflectivity, weaker cost and therefore adequate performance for photovoltaic applications^3–7^. Our study confirms the characteristics of mentioned materials above, which have a band gap range of (0.98 eV to 2.25 eV), an absorption coefficient of 237,311 cm^-1^ (211,556 cm^-1^) and reflectivity of (0.1–0.3%) in the extreme ultraviolet light. The band gap range (1–2.25 eV) and the absorption of extreme ultraviolet light make K_2_SeBr_6_ and K_2_PtBr_6_ as absorber materials in solar cells. These compounds are poor reflector and can be used as an anti-reflection coating material.

The aim of this work is the use of GGA-PBESOL and HSE hybrid approximations to obtain adequate structural, elastic and optoelectronic properties of K_2_XBr_6_ (X = Pt, Se). The paper is organized such as the calculation scheme is detailed in the second part. The exposure and discussion of obtained results are reported in the third section. This work is concluded by an conclusion in the last part.

## Calculation scheme

Calculations were carried out using the DFT framework as implemented in the CASTEP code^8^. The valence states of K_2_XBr_6_(X = Se, Pt) are K: 4s^1^, Se: 4p^4^, Pt: 5d^9^ and Br: 4p^5^. An ultra soft pseudo-potential type Vanderbilt^9^ describes the interaction of valence electrons and ions cores. The GGA-PBESOL of Perdew et al.^10^ is adopted for the non-local correlation exchange effect. The best convergence of the computed structures and energies requires the use of cut-off energy of 630 eV. The irreducible Brillouin zone was sampled up to 8 ×  8 × 8 k-grid on the Monkhorst–Pack scheme^11^. The tolerance of geometry optimization were a difference of total energy 5 × 10^−6^ eV/atom, a maximum ionic Hellmann–Feynman force 10^–2^ eV/Å, maximum stress 2 × 10^–2^ eV/Å^3^ and ionic displacement of 5 × 10^–4^ Å. The calculation of the optical parameters requires the use of uniform distribution of 20 ×  20 × 20 k-points. The self-consistent calculations converge if the total energy is minimal. The structural parameters were estimated using the minimization technique of Broyden-Fletcher-Goldfarb-Shanno (BFGS)^12^, which provide a fast way to find the lowest energy structure. The basic idea behind the hybrid functionals is to mix exchange energies calculated in an exact (Hartree–Fock-like) manner with those obtained from DFT methods in order to improve performance. The accuracy of the electronic properties predicted by density functional theory depends on the used exchange–correlation functional. Non-local hybrid functionals gives more accurate results than semi-local functionals. The non-local Hartree–Fock exchange is an integral part of the hybrid functionals implemented in the FLAPW mehod^13^. The non-local exchange in HSE enlarges the elements of the optical transition matrix and leads to better accuracy of HSE in calculating electronic properties. Omitting the non-local exchange in the transition operator for HSE leads to errors. The importance of non-local correction in the velocity gauge has been widely discussed for non-local pseudo potentials^14,15^. The neglect of the non-local term in the velocity gauge leads to inaccuracy, especially for transitions that involve localized d electrons^16,17^. The non-locality of the potential comes from the fact that the electron Hamiltonian is replaced by an approximate Hamiltonian in the independent electron approximation with an effective potential, which reintroduces the electron–electron interactions in the Kohn–Sham equations.

## Results and discussion

### Structure and morphology

**Figure 1:**
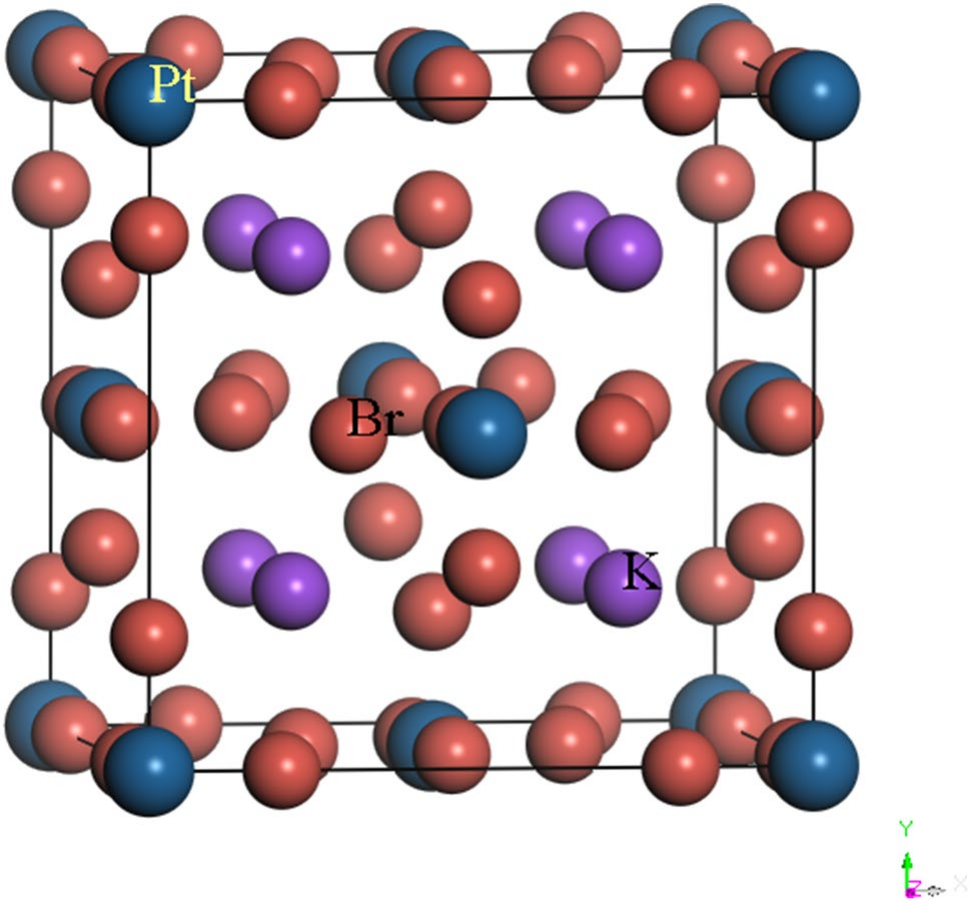
The crystal structure of K_2_PtBr_6_.

**Figure 2:**
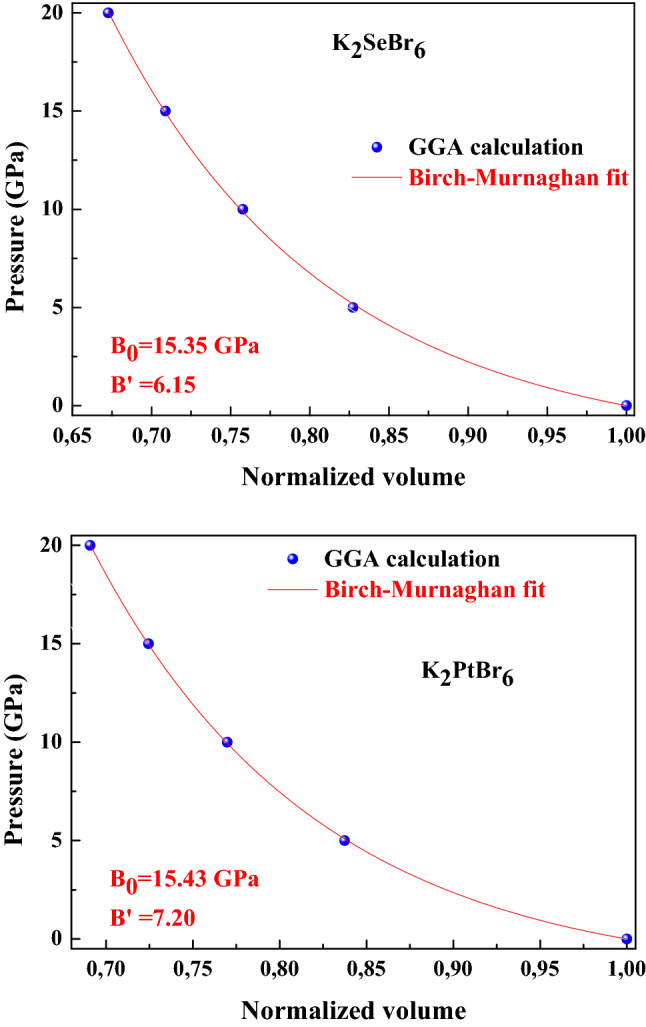
The pressure effect on normalized volume in K_2_SeBr_6_ and K_2_PtBr_6_.

**Figure 3:**
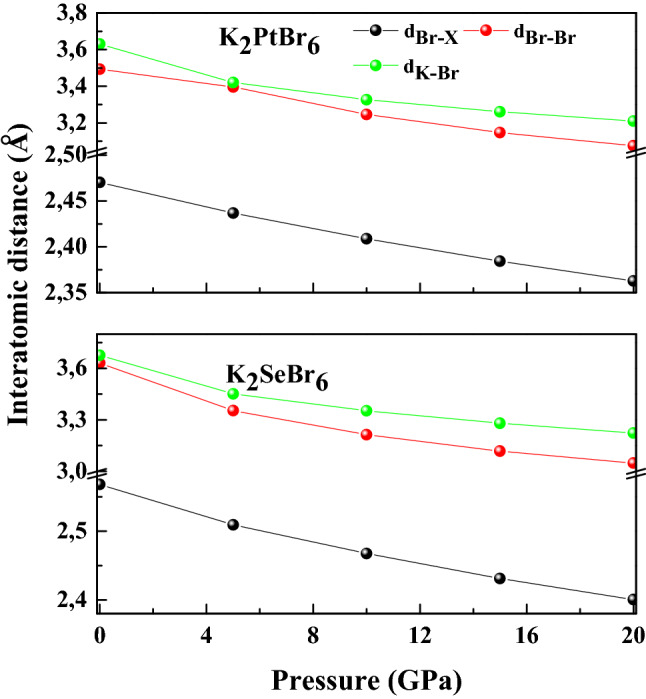
Effect of pressure on bond lengths d_K-Br_, d_Br-Br_ and d_Br-X(X=Se, Pt)_ in K_2_SeBr_6_ and K_2_PtBr_6_.

**Figure 4:**
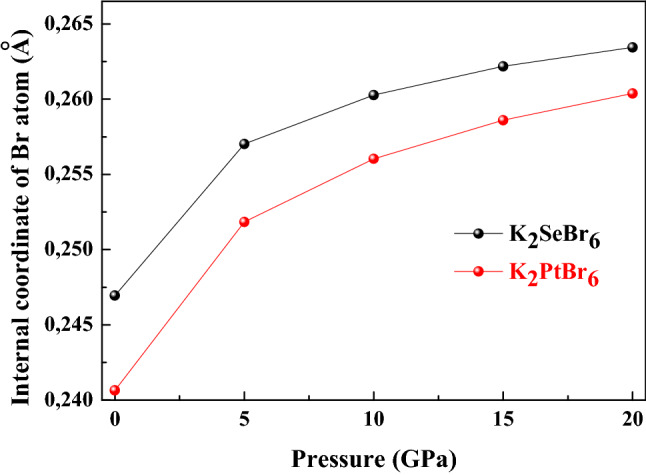
The pressure effect on internal coordinate of Br atom in K_2_SeBr_6_ and K_2_PtBr_6_.

The crystal structure of K_2_PtBr_6_ is illustrated in Fig. 1. The location of atoms is such that (Se, Pt) atom is placed at the center of the octahedron formed by the four atoms of Br. The K atoms occupy interstitial sites. The antifluorite class K_2_XBr_6_(X = Se, Pt) adopt the cubic structure with space group Fm3m at ambient conditions. The occupied Wyckoff sites for K, (Se, Pt) and Br atoms are ± (1/4, 1/4, 1/4) *a*_0_, (0, 0, 0) *a*_0_ and ± (*x*, 0, 0) *a*_0_, ± (0, *x*, 0) *a*_0_ , ± (0, 0, *x*) *a*_0_. The lattice constant, bulk modulus and its pressure derivative of K_2_XBr_6_(X = Se, Pt) are listed in Table 1. The cell constant of K_2_SeBr_6_ and K_2_PtBr_6_ is consistent to the experimental value quoted in the literature^1,2^, where the error is 0.95% and 1%. The bulk modulus calculated for K_2_SeBr_6_ and K_2_PtBr_6_ using the fit scheme P(V/V_0_) as reported in Fig. 2 is in good agreement with available experimental data^1^. The interatomic distances d_X-Br_, d_K-Br_ and d_Br-Br_ in K_2_SeBr_6_ (K_2_PtBr_6_) at equilibrium are 2.5681 Ǻ, 3.6769 Ǻ and 3.6319 Ǻ (2.4703 Ǻ, 3.6306 Ǻ and 3.4936 Ǻ). It should be pointed that, bond lengths reported for K_2_SeBr_6_ are in good agreement with those found in the literature d_Se-Br_ = 2.555 Ǻ, d_Pt-Br_ = 2.50 Ǻ, d_K-Br_ = 3.685 Ǻ and d_Br-Br_ = 3.613 Ǻ^2^. Figure 3 shows the effect of pressure on d_K-Br_, d_Br-Br_ and d_Br-X_ (X = Se, Pt) bond lengths in K_2_SeBr_6_ and K_2_PtBr_6_. The bond lengths in K_2_SeBr_6_ are large than those in K_2_PtBr_6_; hence, the Coulomb interaction in K_2_PtBr_6_ is greater than that in K_2_SeBr_6_, which can be explained by the fact that the distances are inversely proportional to the lattice constant. Also, the distance d_Br-Se_ is greater than that of d_Br-Pt_. All bond lengths decrease monotonously when the pressure increases. Figure 4 displays the effect of pressure on the internal coordinate of Br atom in K_2_SeBr_6_ and K_2_PtBr_6_. The internal coordinate of Br atom in K_2_PtBr_6_ is lower than that of the same atom in K_2_SeBr_6_, and this is explained by the fact that it is inversely proportional to the atom radius of Se (1.15 Ǻ) and Pt (1.35 Ǻ).

**Table 1:** The lattice constant, bulk modulus and its pressure derivative and elastic moduli of K_2_XBr_6_(X = Se, Pt).

	K_2_SeBr_6_			K_2_PtBr_6_		
	This Work	Experiment	Other	This Work	Experiment	Other
A (Å )	10.2653	10.363 ^1^		10.3995	10.293 ^1^	
*x*	0.24065			0.24695		
*B_0_ (GPa)	15.35	16.70 ^1^		15.43	15.20 ^1^	
B’	6.15			7.20		
C_11_ (GPa)	15.49	23.20 ^1^		22.27	21.60 ^1^	
C_12_ (GPa)	11.95	13.50 ^1^		8.97	12.0 ^1^	
C_44_ (GPa)	7.08	9.30 ^1^		5.95	8.50 ^1^	
**B_0_ (GPa)	13.13			13.40

### Elastic constants and related parameters

The knowledge of elastic constants is essential for a better theoretical understanding of the properties of materials that are determined by the phonons density of states and the electron–phonon interaction processes. The three independent elastic constants C_11_, C_12_ and C_44_ require for their elastic characterization. The elastic moduli of K_2_XBr_6_(X = Se, Pt) computed at equilibrium using GGA-PBESOL are reported in Table 1. No theoretical value is reported in the literature, then our computation is prediction. The elastic constants of K_2_SeBr_6_ and K_2_PtBr_6_ are significantly small because of their quite large reticular distances, low Colombian forces, so they are quite soft and tolerant to damage. This result is qualitatively explained in these two materials by their binding forces, which are mainly ionic. It was noted that C_11_, C_12_ and C_44_ agree reasonably with their experiment values^1^. The elastic stability of K_2_SeBr_6_ and K_2_PtBr_6_ was defined taking into account the Born’s criteria, from which the following conditions must be satisfied for zinc blend structure^18^:

1 $$ 0\langle C_{11} + 2C_{12} ,\;0\langle C_{44} ,\;0\langle C_{11} - C_{12} ,\;C_{12} \langle C_{11} $$

**Figure 5:**
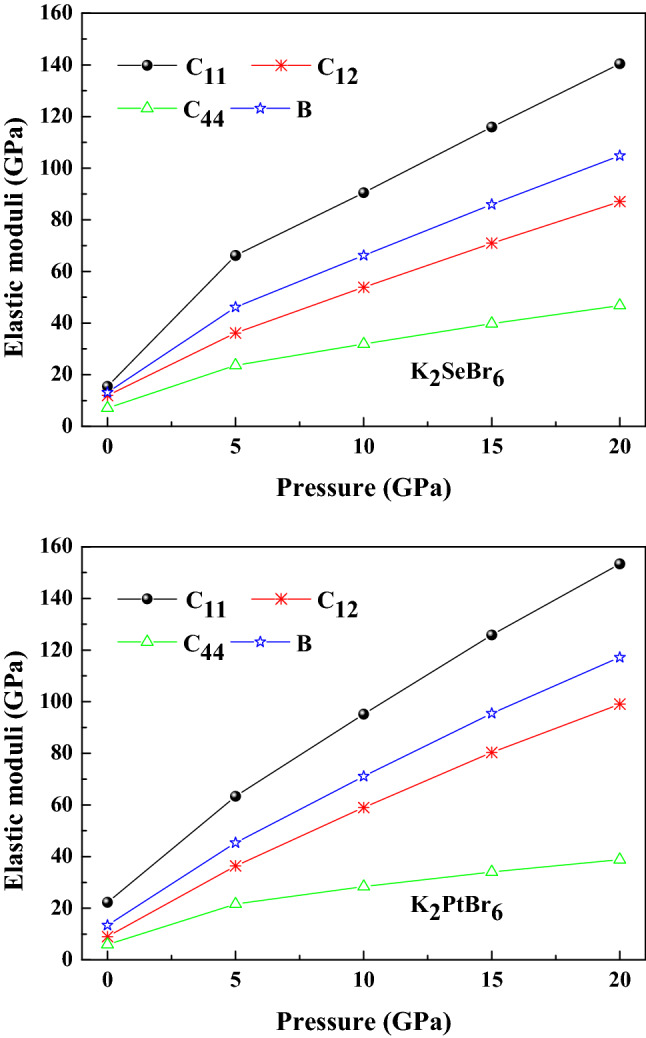
Elastic moduli of K_2_SeBr_6_ and K_2_PtBr_6_.

**Figure 6:**
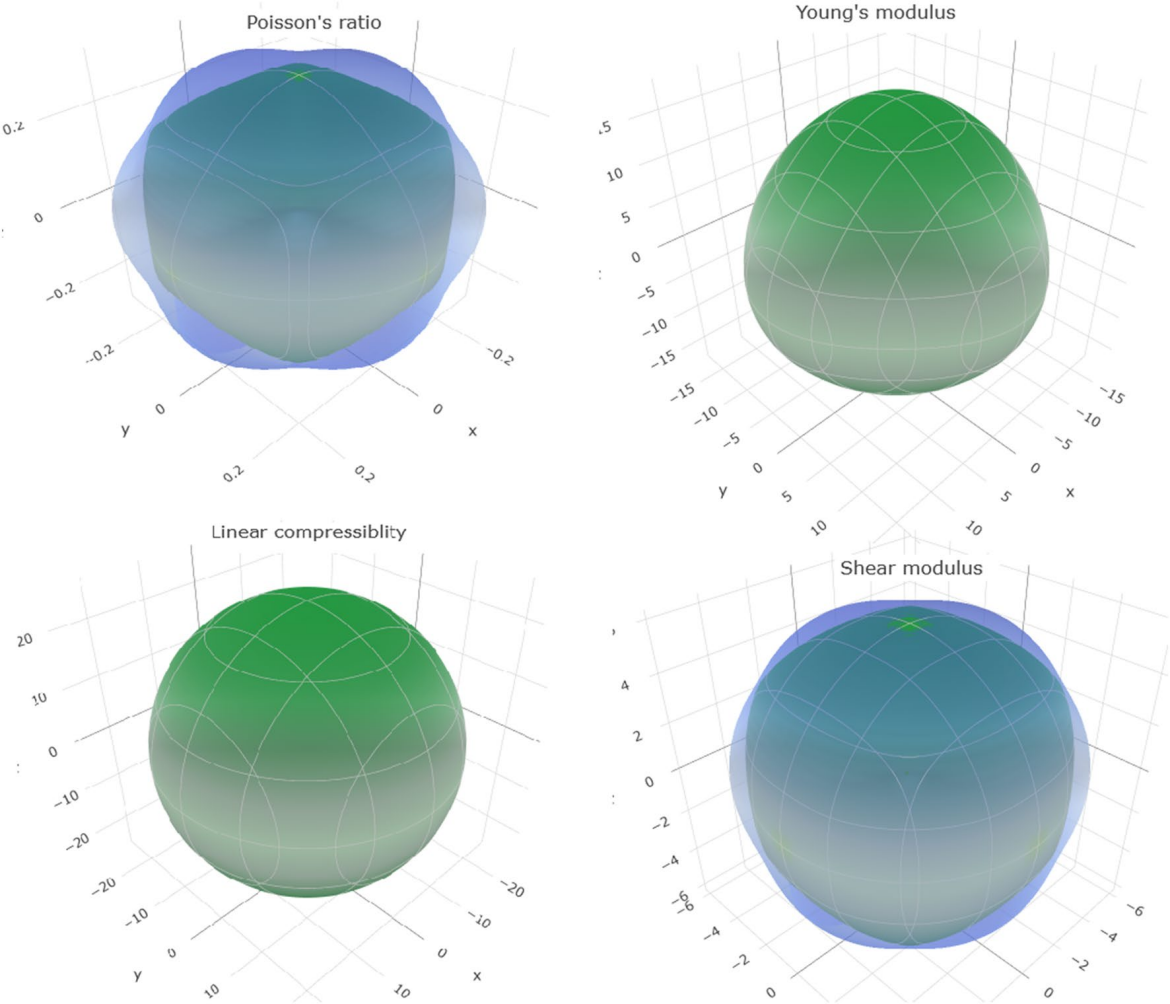
The orientation effect on Poisson's ratio, Young's modulus, linear compressibility and shear modulus in K_2_PtBr_6_.

The bulk modulus calculated from the elastic constants is identical to that deduced from equation of state fitting P(V/V_0_). This makes our results as reliable. Figure 5 visualizes the dependence on pressure of K_2_XBr_6_(X = Se, Pt) elastic moduli. It is observed that the elastic values of GGA-PBESOL increase as a function of the applied pressure, from zero to 20 GPa. These compounds show weaker elastic constants, which explain their lower hardness. The bulk modulus, shear modulus, Young’s modulus, Poisson’s ratio, the universal anisotropy and B_H_/G_H_ ratio for isotropic polycrystalline materials of K_2_XBr_6_(X = Pt, Se) using theVoigt-Reuss-Hill approximation^19–21^ are reported in Table 2. The values of the Poisson coefficient between 0.25 and 0.5 are associated with the interatomic forces of central types and covalent bonding character. The nature of the bonds in a compound is described by the factor σ, either ionic-covalent (0.16 ≤ σ ≤ 0.30) in K_2_PtBr_6_ (0.29) and metallic (σ ≥ 0.33) in K_2_SeBr_6_ (0.35). The Pugh’s criterion (B_*H*_/G_*H*_) and universal anisotropy indicate that K_2_XBr_6_(X = Pt, Se) are ductile and anisotropic. The extreme values of Young’s modulus, linear compressibility, shear modulus and Poisson’s ratio for K_2_XBr_6_(X = Pt, Se) are listed in Table 3. These values prove the isotropic linear compressibility and confirm the anisotropy of the other parameters and the anisotropy is more pronounced in K_2_SeBr_6_. We represent in Fig. 6 using ELATE software^22^ the effect of orientation on mechanical parameters for K_2_PtBr_6_. Young’s modulus, shear modulus and Poisson's ratio are anisotropic, while linear compressibility is isotropic.

**Table 2:** The extreme values of Young’s modulus (GPa), linear compressibility (GPa), shear modulus (GPa) and Poisson’s ratio for K_2_XBr_6_(X = Pt, Se).

Material	Young’smodulus		Linear compressibility		Shear modulus		Poisson’s ratio	
	*E*_min_	*E*_max_	^*β*^min	^*β*^max	*G*_min_	*G*_max_	^*σ*^ min	^*σ*^ max
K_2_SeBr_6_	5.0801	18.014	25.374	25.374	1.7694	7.0839	0.22305	0.94375
K_2_PtBr_6_	15.565	17.117	24.857	24.857	5.9567	6.6484	0.26729	0.33683

**Table 3:** The bulk modulus, shear modulus, Young’s modulus, Poisson’s ratio, anisotropy factorand B_H_/G_H_ratio for K_2_XBr_6_(X = Pt, Se).

Material	B (GPa)			(GPa)			E_H_ (GPa)	σ_H_	_A_U	B_H_/G_H_
	B_V_	B_R_	B_H_	G_V_	G_R_	G_H_				
K_2_SeBr_6_	13.137	13.137	13.137	4.9581	3.2179	4.088	11.111	0.35903	2.70401	3.21
K_2_PtBr_6_	13.41	13.41	13.41	6.2334	6.2154	6.2244	16.171	0.29902	0.0145	2.15

### Band structure and states densities

**Figure 7:**
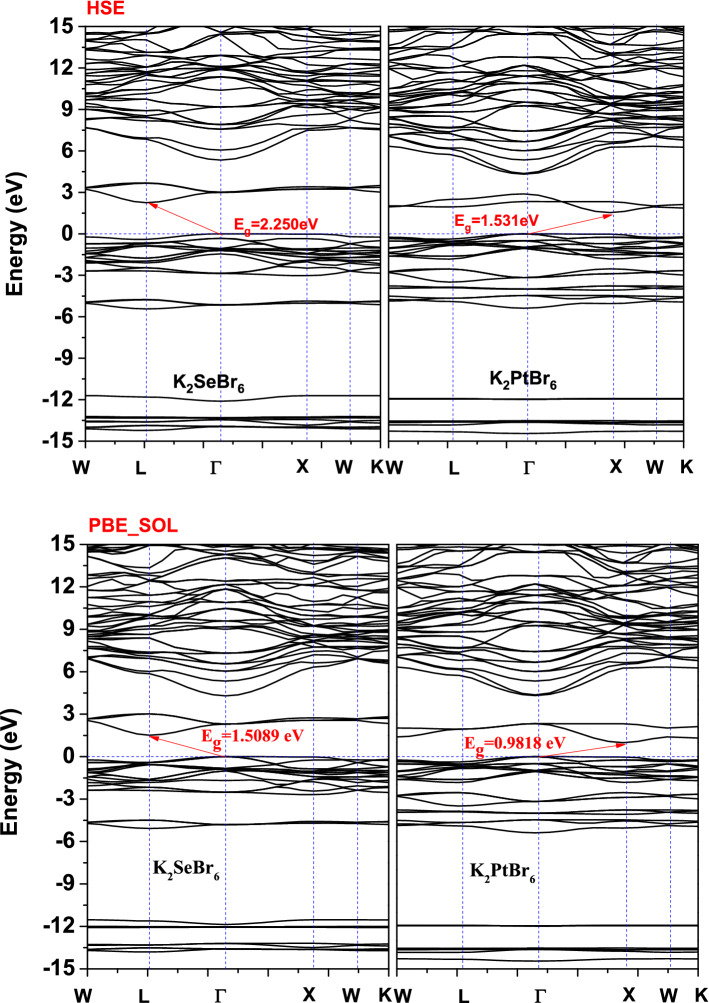
Band structures of K_2_XBr_6_(X = Pt, Se) in zinc blend structure performed with GGA (PBE-SOL) and HSE hybrid.

**Figure 8:**
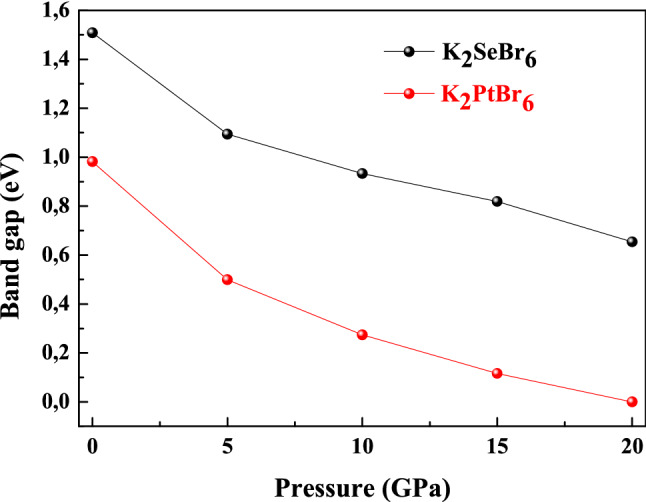
Fundamental band gap of K_2_XBr_6_(X = Pt, Se) with GGA (PBE-SOL) and HSE hybrid.

**Figure 9:**
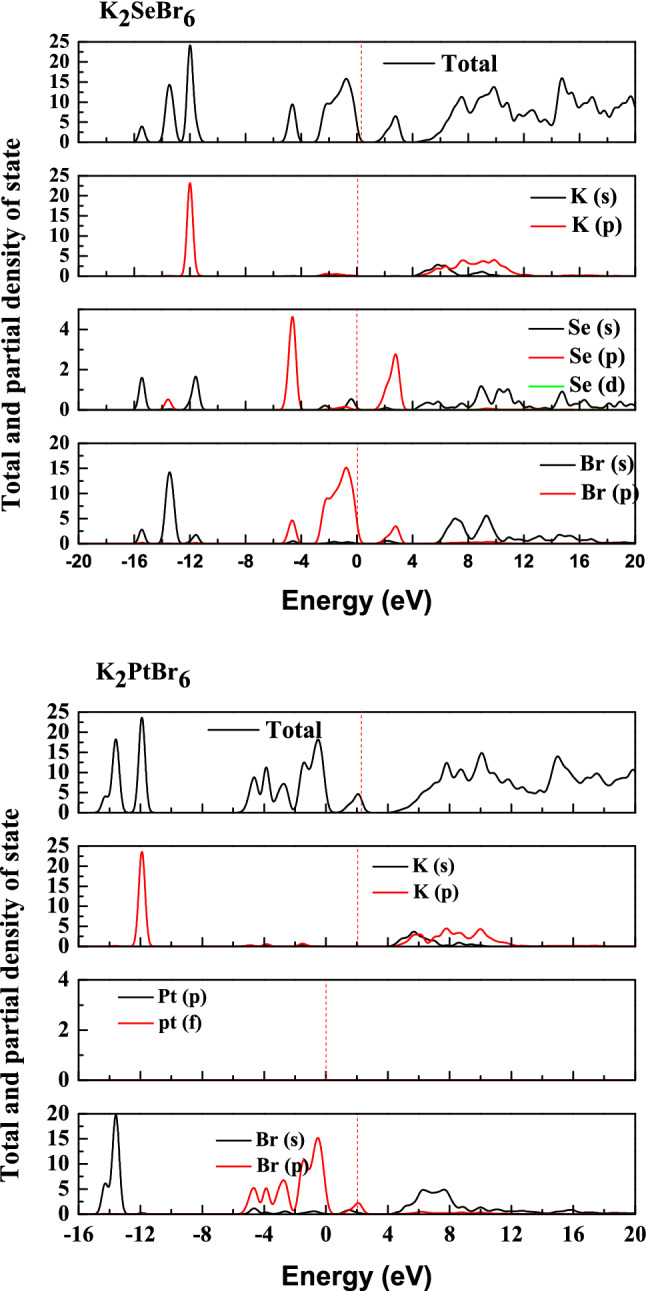
Total and partial density of states for K_2_SeBr_6_ and K_2_PtBr_6_.

Understanding the band structure and estimating band gap of K_2_XBr_6_(X = Pt, Se), we use both GGA (PBE-SOL) and HSE hybrid functional as shown in Fig. 7. The calculations were conducted on K_2_XBr_6_(X = Pt, Se), by neglecting the presence of the K-state in the (Pt, Se) site. The electronic band structure of K_2_SeBr_6_ and k_2_PtBr_6_ were computed using the equilibrium lattice constant. The bottom of the conduction band is at Γ point for K_2_SeBr_6_ and k_2_PtBr_6_. The top of the valence band is at L and X points in K_2_SeBr_6_ and k_2_PtBr_6_ compounds, which indicate an indirect band gap Γ-L (Γ-X) of 1.5089 eV and 2.250 eV (0.9818 eV and 1.531 eV) for K_2_SeBr_6_ (k_2_PtBr_6_). No experimental and theoretical value are present in the literature, and then our results are predictions. Note that the HSE approximation gives a value close to the experimental one. We report the various band gaps at equilibrium lattice constant for K_2_SeBr_6_ and k_2_PtBr_6_ using GGA and HSE in Table 4. By varying the applied pressure between 0 and 20 GPa, the fundamental band gap as shown in Fig. 8 decreases. K_2_PtBr_6_ becomes metallic at a pressure of 15 GPa. We visualize the plots of PDOS and TDOS of K_2_SeBr_6_ and K_2_PtBr_6_ in Fig. 9. The top of valence band region is − 2.86 eV to E_F_ (− 2 eV to E_F_) for K_2_SeBr_6_ (k_2_PtBr_6_). The electronic contribution in this region is due mainly to Br: p orbital in K_2_SeBr_6_ and K_2_PtBr_6_. The first conduction band of K_2_SeBr_6_ (K_2_PtBr_6_) starts at 1.68 eV (4 eV), then the transitions occur between Br: p and K: p sites. It is noted that the Pt site does not participate in the electronic contribution at the conduction and valence bands.

**Table 4:** The various band gaps at zero pressure for K_2_SeBr_6_ and K_2_PtBr_6_.

Material	EΓ-Γ	EΓ-X	EΓ-L	EX-X	EL-L
K_2_PBr_6_					
E_0_ (eV)	2.323	0.98	1.88	1.14	2.31
α × 10^–2^ (eV/GPa)	− 9.94				
β × 10^–3^ (eV/GPa^2^)	2.8				
K_2_SeBr_6_					
E_0_ (eV)	2.29	2.56	1.5	2.58	1.902
α × 10^–2^ (eV/GPa)	− 7.09				
β × 10^–3^ (eV/GPa^2^)	1.56

### Optical properties

The real dielectric constant is a measure of polarization, while the imaginary part is a measure of the dielectric losses. The complex dielectric function is the sum of real and imaginary parts.

$$ \varepsilon \left( \omega \right) = \varepsilon_{1} \left( \omega \right) + i\varepsilon_{2} \left( \omega \right) $$

The optical quantities such as reflectivity, absorption, loss function and refractive index depend on the structure of the material. These parameters cited above are isotropic in a material with cubic structure. The reflectivity of any material is calculated by dielectric function through the equation:

2 $$ R\left( \omega \right) = \left| {\frac{{\left( {\varepsilon_{1} } \right)^{1/2} - 1}}{{\left( {\varepsilon_{1} } \right)^{1/2} + 1}}} \right| $$

**Figure 10:**
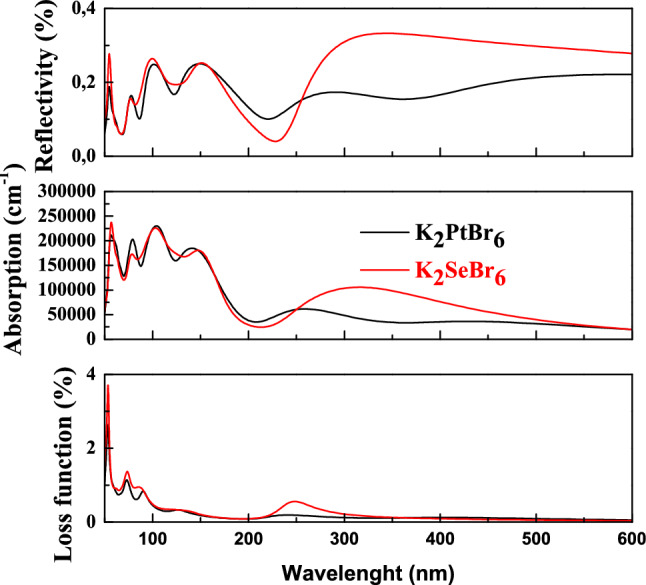
The reflectivity, absorption and loss function in K_2_SeBr_6_ and K_2_PtBr_6_.

We display the plots of reflectivity, absorption and loss function as a function of wavelength for K_2_SeBr_6_ and K_2_PtBr_6_ in Fig. 10. The reflectivity is a measure of the ability of a material to reflect radiation. The reflectivity of K_2_SeBr_6_ and K_2_PtBr_6_ starts at wavelength around 60 nm and reaches several peaks of maxima (0.23) and minima (0.05) in the field of extreme ultraviolet light. In practice, the roughness, uniformity of thickness, inter diffusion, oxidation and thermal stability limit the reflectivity. We observe various absorption peaks in extreme ultraviolet light. These peaks are due to the electronic transitions from the top of the valence band to the bottom of the conduction band. The maximum absorption is between 234,720 cm^-1^ and 229,405 cm^-1^ at wavelength range 56 nm to 105 nm for K_2_SeBr_6_ and K_2_PtBr_6_. Indeed, K_2_SeBr_6_ and K_2_PtBr_6_ have a narrow gap and absorb extreme ultraviolet light and consequently, they are candidates in the fields of photo catalysis and photovoltaic. The loss function is calculated through the equation:

3 $$ L\left( \omega \right) = \frac{{\varepsilon_{2} \left( \omega \right)}}{{\varepsilon_{1}^{2} \left( \omega \right) + \varepsilon_{2}^{2} \left( \omega \right)}} $$

**Figure 11:**
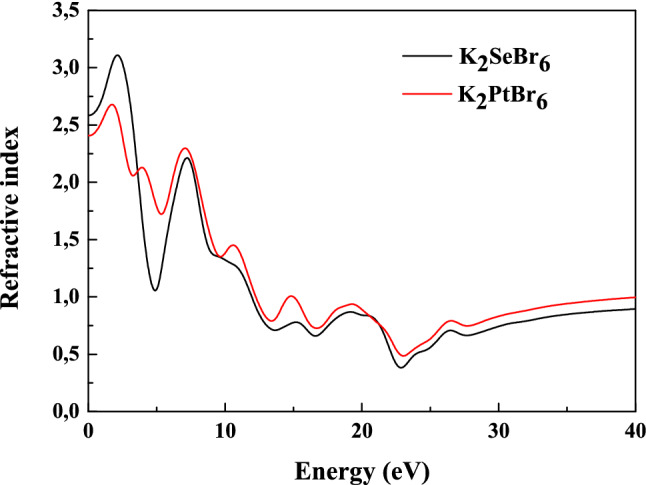
The refractive index in K_2_SeBr_6_ and K_2_PtBr_6_.

The loss function demonstrates the existence of two major plasmonic processes, with intensity 3.7 and 1.35 located around 53.5 nm and 72.8 nm. There is no loss in the ultra violet and visible light domains. We present in Fig. 11 the refractive index of K_2_SeBr_6_ and K_2_PtBr_6_ as a function of energy. The static refractive index is 2.583 (2.407) for K_2_SeBr_6_ (K_2_PtBr_6_). It reaches a series of maxima 3.106 (2.678) and minima 0.383 (0.485) between 1.7 eV and 23 eV for K_2_SeBr_6_ (K_2_PtBr_6_). It is reported that an experimental refractive index 2.15 and 2.11 for K_2_SeBr_6_ and K_2_PtBr_6_ calculated by N. Wruk et al.^1^. The refractive index is given as:

4 $$ n\left( \omega \right) = \frac{\sqrt 2 }{2}\left[ {\varepsilon_{1} + \sqrt {\varepsilon_{1}^{2} + \varepsilon_{2}^{2} } } \right] $$

**Figure 12:**
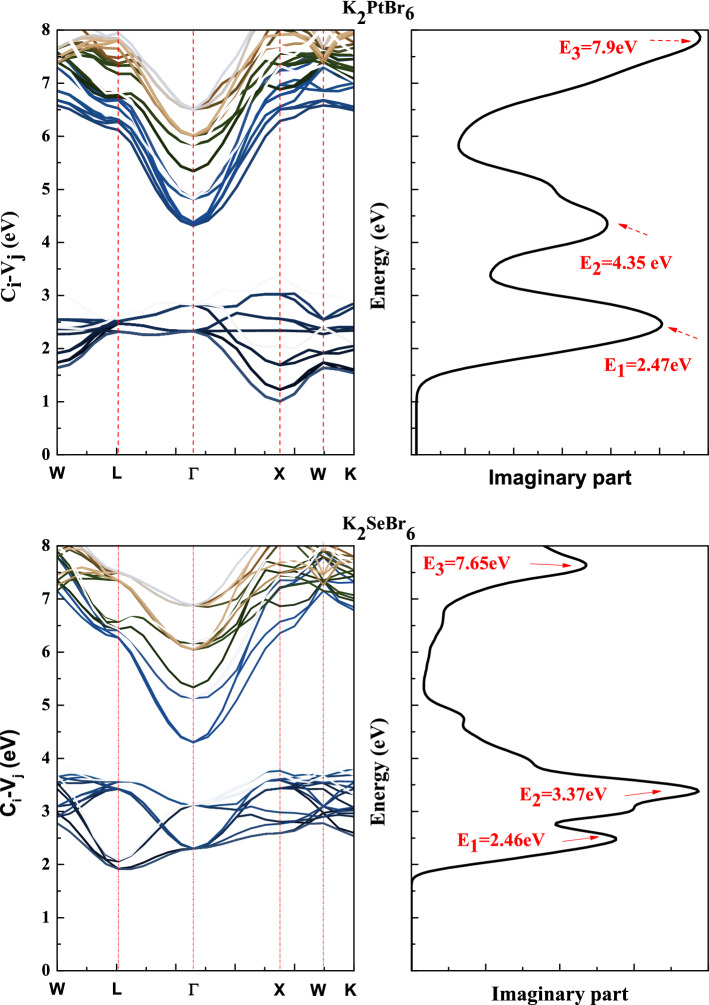
The imaginary part and transition energy in K_2_SeBr_6_ and K_2_PtBr_6_.

The refractive index is more important when photons move through the material and when bonds between atoms are covalent. The static refractive index enhanced with the expansion of the electronic cloud and the increase in density on the structure. The general trend is that the decrease in reflectivity results from the increase in absorption and the decrease in refractive index. The plots of imaginary part and $E(k) = E_{Cj} (k) - E_{Vi} (k)$ for K_2_SeBr_6_(X = Se, Pt) are reported in Fig. 12 (right and left panel). The imaginary part and optical transitions are connected to the absorption coefficient. The main contribution to the optical transitions from six top valence bands to seven lower conduction bands for K_2_SeBr_6_(X = Se, Pt) are reported in Table 5. The isotropic optical parameters of K_2_SeBr_6_ and K_2_PtBr_6_ makes them as windows and lenses. The band gap range (1–2.25 eV) and absorption of extreme ultraviolet light make K_2_SeBr_6_ and K_2_PtBr_6_ as absorber materials.

**Table 5:** The main contribution to the optical transitions for K_2_SeBr_6_(X = Se, Pt).

Peaks	*W* → *L*	*L* → Γ	Γ → *X*	*X* →*W*	*W* → *K*
**K**_**2**_**PtBr**_**6**_					
*E* = 2.47* eV*	*V*_5_ → *C*_2_, *V*_*3*_ → *C*_2_ *V*_4_ →*C*_1_, *V*_6_ → *C*_1_		*V*_5_ → *C*_1_	*V*_3_ → *C*_2_	
*E* = 4.35* eV*		*V*_3_ → *C*_3_, *V*_1_ → *C*_4_, *V*_1_ → *C*_3_			
*E* = 7.9* eV*	*V*_3_ → *C*_4_, *V*_4_ → *C*_4_ *V*_5_ → *C*_4_	*V*_6_ → *C*_7_, *V*_6_ → *C*_5_ *V*_5_ → *C*_7_, *V*_5_ → *C*_6_	*V*_6_ → *C*_7_, *V*_5_ → *C*_7_ *V*_6_ → *C*_6_, *V*_3_ → *C*_6_ *V*_3_ → *C*_5_, *V*_1_ → *C*_6_		
*E* = 7.47* eV*	*V*_6_ → *C*_7_		*V*_6_ → *C*_7_, *V*_4_ → *C*_7_ *V*_3_ → *C*_7_		
**K**_**2**_**SeBr**_**6**_					
*E* = 2.46* eV*	*V*_1_ → *C*_1_, *V*_6_ → *C*_1_, *V*_3_ → *C*_1_	*V*_5_ → *C*_1_, *V*_1_ → *C*_2_ *V*_3_ → *C*_3_	*V*_1_ → *C*_1_		
*E* = 3.35* eV*	*V*_3_ → *C*_3_, *V*_5_ → *C*_1_, *V*_1_ → *C*_3_, *V*_3_ → *C*_3_	*V*_5_ → *C*_2_, *V*_3_ → *C*_6_, *V*_3_ → *C*_4_	*V*_6_ → *C*_1_	*V*_3_ → *C*_2_	
*E* = 7.65* eV*	*V*_5_ → *C*_3_, *V*_6_ → *C*_6_ *V*_6_ → *C*_7_, *V*_4_ → *C*_6_ *V*_5_ → *C*_7_	*V*_6_ → *C*_7_, *V*_6_ → * C*_5_ *V*_5_ → *C*_7_, *V*_6_ → *C*_4_	*V*_6_ → *C*_7_, *V*_3_ → *C*_7_ *V*_6_ → *C*_5_, *V*_2_ → *C*_7_ *V*_6_ → *C*_5_	*V*_6_ → *C*_4_, *V*_5_ → *C*_2_	

## Conclusion

Employing a plane-wave pseudo-potential using the DFT framework, within the generalized gradient approximation, we studied the structural, mechanical and optoelectronic parameters of K_2_PtBr_6_ and K_2_SeBr_6_ hexahalometallate materials. The bulk modulus of K_2_SeBr_6_ and K_2_PtBr_6_ agrees well with experiment value where the error is 8% and 1.4%. The elastic constants of K_2_SeBr_6_ and K_2_PtBr_6_ are significantly smaller, then they are fairly soft and damage tolerant. An electronic study shows that K_2_PtBr_6_ is indirect band gap semiconductor and becomes metallic at a pressure of 15 GPa. The partial density of states indicates that the valence electrons are transferred from Br: p state to K: p site. The band gap size, optical absorption and reflectivity make K_2_SeBr_6_ and K_2_PtBr_6_ as candidate absorbers. The static refractive index increases with the expansion of the electronic cloud and the increase in density on the structure. The general trend is that the decrease in reflectivity results from the increase in absorption and the decrease in refractive index. There is no loss in the ultra violet and visible light domains. The compounds are poor reflector and can be used as an anti-reflection coating material.
